# 829 Biosynthetic Wound Matrix Applied to Autologous Skin Cell Suspension-treated Burns Supports Good Outcomes

**DOI:** 10.1093/jbcr/iraf019.360

**Published:** 2025-04-01

**Authors:** Tait Olaveson

**Affiliations:** The Burn Center at Eastern Idaho Regional Medical Center

## Abstract

**Introduction:**

Autologous Skin Cell Suspension (ACSC) application has been shown to facilitate closure and epithelization of deep partial and partial thickness burn and trauma wounds. Optimal dressing over ACSC has not been well established. Options have included nonabsorbent, non-adherent dressings and silver-impregnated antimicrobial dressings.

A temporary biosynthetic wound matrix (BWM) consisting of a bilayer of a thin outer silicone and an inner layer of nylon coated with collagen and aloe vera has not been traditionally used or evaluated for efficacy over ASCS. BWM has several notable benefits: 1) it is transparent allowing for wound assessment during healing; 2) it quickly adheres to a wound and only requires a single application; 3) it can be applied with different tensions to control the porosity and overall moisture management.

**Methods:**

Patients were selected after usual burn and wound preparation and optimized for ASCS application including debridement and removal of burn eschar with application of cadaver allograft. Surveillance cultures were obtained and treated if indicated. Once the wound bed was deemed ready for ASCS, patients were selected for ACSC with BWM, ACSC + split thickness skin graft (STSG) with BWM, and if both extremities were available, an alternate dressing was applied and treated in usual post grafting protocol.

BWM care post application included dry gauze vs diluted bleach ringout with daily dressing changes. Time of removal of BWM was included. Post-surgical imaging was obtained and a blinded burn surgeon evaluated images and scored as an equal, superior or inferior outcome based on graft outcome appearance. Burns included deep partial second-degree, and third-degree burns.

**Results:**

Patients showed no inferiority with BWM compared to any laterally contrasted dressing. Patients treated with ACSC and BWM alone showed high percentage of healing and graft take. Burns treated with BWM showed decrease inflammation with no signs of hypertrophy. This was more apparent in third degree burns treated with ACSC and STSG with BWM. Patient subjectively scored less pain and better cosmetic outcome with BWM dressings. Long-term follow up is being conducted to determine if outcomes remain beneficial compared to contrast dressings.

**Conclusions:**

BWM was not inferior to any contrasting dressing and has potential benefits. Limitations include low number treated which is inherent to a limited case series. Long term follow up is ongoing, but results are favorable.

**Applicability of Research to Practice:**

This data provides evidence for best treatment algorithm when choosing dressings for ASCS-treated burns.

**Funding for the Study:**

N/A

